# Autotransplantation of the Spleen Following Atraumatic Splenic Rupture Secondary to Infectious Mononucleosis: A Case Report

**DOI:** 10.7759/cureus.76429

**Published:** 2024-12-26

**Authors:** Jack Menzie, Niyaz Naqash

**Affiliations:** 1 Department of General Surgery, Monash Health, Melbourne, AUS; 2 Department of Upper Gastrointestinal and Hepatobiliary Surgery, Monash Health, Melbourne, AUS

**Keywords:** atraumatic splenic rupture, autotransplantation, epstein-barr virus, general surgery, infectious mononucleosis (im), laparotomy, rare cause of acute abdominal pain, rupture of the spleen, spleen-preserving surgery, splenectomy

## Abstract

Atraumatic splenic rupture (ASR) is a rare and life-threatening condition that presents diagnostic difficulties due to its rarity and non-specific clinical symptoms. It often requires computed tomography (CT) imaging for accurate diagnosis and surgical planning. Splenectomy is the standard treatment for unstable patients, but autotransplantation of splenic tissue may reduce the lifelong risks of overwhelming post-splenectomy infections (OPSI) by preserving some immunological function. However, autotransplantation is rarely performed in ASR due to the delicate nature of the spleen and the presence of underlying pathology.

This report describes a 27-year-old male with unstable ASR secondary to Epstein-Barr virus (EBV) infection, a condition linked to splenomegaly and rupture in 0.1-0.5% of cases. The patient underwent emergency splenectomy and autotransplantation of viable splenic tissue into the omentum. Despite the challenges associated with autotransplantation in ASR, the procedure was successful, with the patient achieving excellent recovery and remaining complication-free at the 12-month follow-up. This case illustrates the potential role of autotransplantation in ASR and highlights the need for further research to refine management strategies and improve outcomes.

## Introduction

Atraumatic splenic rupture (ASR) is a rare but life-threatening condition characterized by spontaneous splenic rupture without external trauma ​[1].​ Unlike splenic rupture caused by blunt abdominal injury, ASR typically arises from pathological changes within the spleen or systemic diseases ​[2]​. Common causes of ASR include infections like Epstein-Barr virus (EBV), malignancies, metabolic disorders, vascular conditions, and hematological abnormalities ​[2]​. 

EBV is a commonly acquired infection, with up to 90% of individuals infected by age 30 ​[3].​ While often asymptomatic in children, young adults may develop infectious mononucleosis (IM), presenting with symptoms such as abdominal pain, nausea, vomiting, odynophagia, myalgia, muscle weakness, headaches, and papular rash, which can make diagnosis difficult ​[3,4]​. IM is a self-limiting lymphoproliferative disorder that can lead to splenomegaly during its active phase increasing the risk of rupture ​[3,5].​ Splenic rupture occurs in approximately 0.1-0.5% of IM cases, often triggered by actions like sneezing, coughing, or vomiting, which apply pressure to the fragile spleen ​[6,7]​. 

Although rare, timely recognition of ASR is crucial due to its mortality rate of up to 12.2% ​[8,9]​. Management of ASR depends on the patient’s hemodynamic status, following blunt splenic trauma guidelines. Stable patients may be managed conservatively, while unstable patients often require splenectomy ​[9]​. Splenectomy patients have a lifetime risk of developing overwhelming post-splenectomy infections (OPSI)​ [10]​. Autotransplantation of splenic tissue may reduce this risk by preserving some immunological function, but studies have focused primarily on elective and trauma cases as ASR often results in significant spleen destruction ​[10,11]​. 

The presented case highlights a patient with unstable ASR secondary to EBV, successfully treated with a total splenectomy and reimplantation of splenic tissue in the omentum, resulting in excellent recovery and no complications over 12 months. 

## Case presentation

A 27-year-old male presented to the emergency department with a two-day history of severe right lower quadrant pain, nausea, anorexia, diarrhea, and difficulty passing urine, on a background of three days of sore throat and rhinorrhea. His past medical history included epilepsy, for which he took levetiracetam. Otherwise, there was no significant medical or surgical history. All family members were well, and there was no recent history of travel or trauma. The patient's vitals were borderline stable, with a blood pressure of 119/86 mmHg, SpO2 of 97%, and a respiratory rate of 22. However, he was showing signs of decompensation, with a heart rate of 117 bpm and a temperature of 38.1°C. On examination, his abdomen was distended but otherwise soft; he was peritonitic with rebound tenderness in the right lower quadrant. 

**Figure 1:**
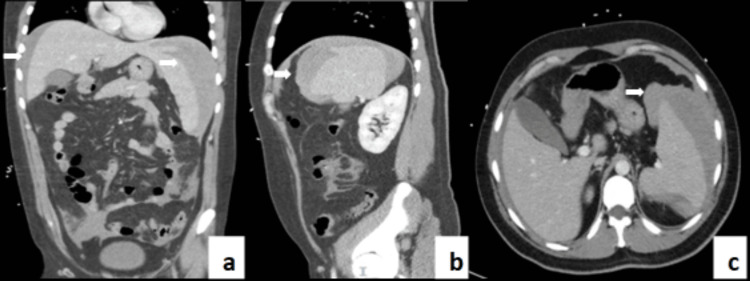
CT images (a. coronal, b. sagittal, c. axial) showing a large volume of hemoperitoneum and the site of splenic rupture White arrows indicate hemoperitoneum. CT, computed tomography

Preliminary blood tests showed elevated white blood cell count, inflammatory markers, and liver function tests (Table 1). A subsequent IM screen was performed, which was positive, suggesting active IM. An urgent contrast-enhanced computed tomography (CT) scan was arranged. The CT (Figure 1) revealed a large volume of hemoperitoneum in the perihepatic, right colic gutter, perisplenic, and left colic gutter regions, likely due to a splenic laceration at the superior margin.

**Table 1:** Patient pathology results compared to normal reference ranges

Pathology Test and Unit	Patient Value	Normal Reference Range
Hemaglobin (Hb) g/L	109	130-180
White Cell Count (WCC)/L	30.7	4-11
Neutrophils/L	10.3	2-8
Lymphocytes/L	17.8	1-4
Monocytes/L	2.6	2-1
Alkaline Phosphatase (ALP) U/L	174	30-110
Alanine Aminotransferase (ALT) U/L	579	5-40
Gamma Glutamyl Transferase (GGT) U/L	97	5-50
Bilirubin mcmol/L	27	0-20
International Normalized Ratio (INR)	1.2	0.8-1.2
C-Reactive Protein (CRP) mg/L	37	0-5

**Figure 2:**
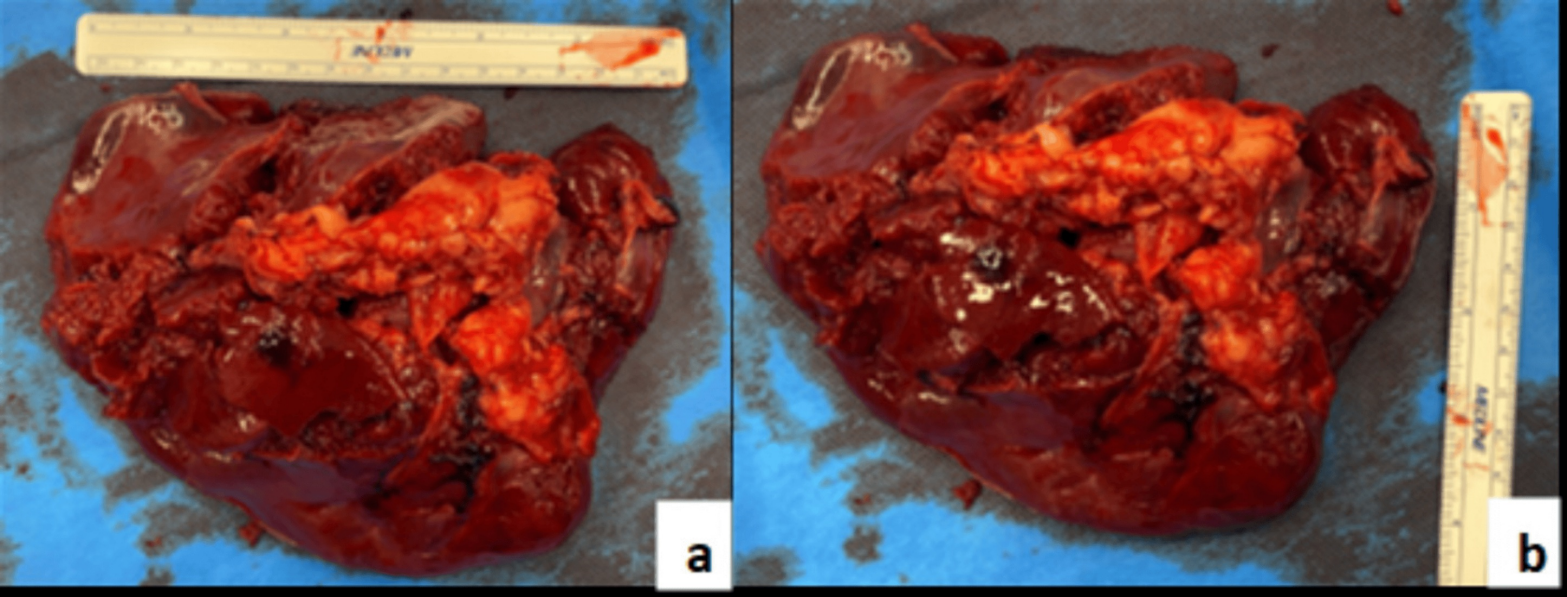
Excised ruptured spleen with friable tissue in multiple areas (a. Measure in width; b. Measure in length)

**Figure 3:**
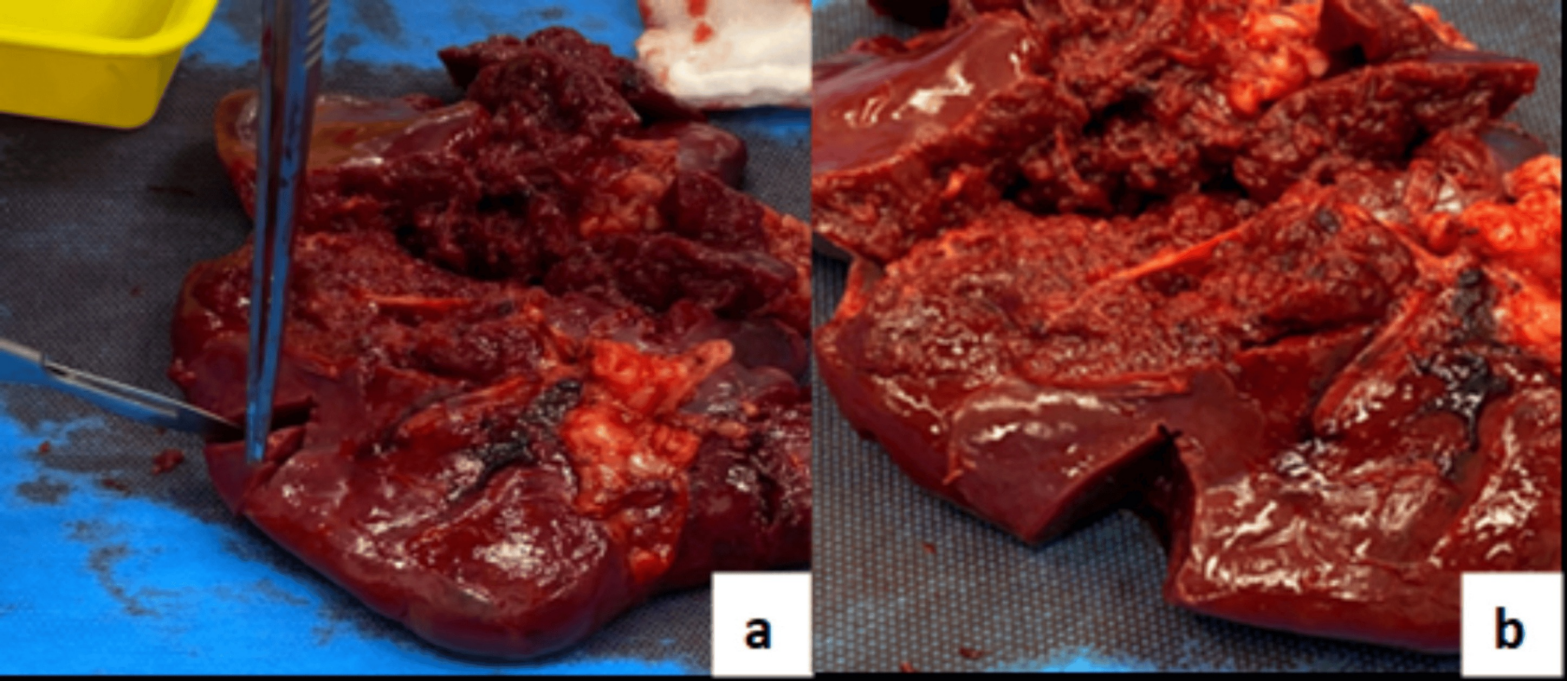
a. Small wedge of healthy spleen excised by the surgeon. b. Spleen after excision of the small healthy wedge

The patient proceeded directly to the theatre for an emergency surgery via laparoscopic approach. Upon inspection after Hasson port entry, a large volume of blood and clots was observed. Compression with Ray-Tec sponges was attempted, and entry to the lesser sac via the greater omentum was made to access the hilar vessels. However, due to poor visualization and ongoing bleeding, the decision was made to convert to laparotomy. An upper midline incision was made just below the umbilicus, and the peritoneum was carefully entered. Lateral mobilization was then performed using Ligasure on the phrenocolic and splenocolic ligaments. The gastroplenic and lienorenal ligament vessels were controlled with Ligasure and 3-0 Prolene sutures, with preservation of the stomach and pancreatic tail. The spleen was completely excised as it was found to be friable in multiple areas (Figure 2) and measured 15x11 cm. A small wedge of the healthy spleen (Figure 3) was identified and autotrasplanted into the omentum. Lavage of the peritoneal cavity was performed with normal saline until the fluid cleared, and Tisseel was applied to the cavity. Two drains were placed: one in the pelvis and one in the left upper quadrant. The abdomen was then closed, and the patient was transferred to the ICU. His estimated intraoperative blood loss was 3-4 L, which was replaced with blood transfusions during the procedure.

Post-surgery, the patient had a mostly unremarkable hospital course and progressed well on the wards after being extubated on postoperative day one. He was started on antibiotics, received the appropriate splenectomy vaccinations, and was enrolled in the spleen registry. Once the patient's drains were removed and he was tolerating a normal diet, he was discharged on day seven post-operation. Subsequent follow-ups occurred at one month and 12 months, with the patient reporting he was doing well and denied any pain, infections, or postoperative complications.

## Discussion

ASR is a rare and life-threatening condition often associated with underlying pathologies such as EBV infection [1,2]. Occurring in only 0.1-0.5% of cases, the limited incidence has resulted in a scarcity of documented cases in the literature [7,12]. This rarity, combined with the absence of preceding trauma and the broad differential diagnoses for abdominal pain, poses significant diagnostic challenges for clinicians [8,12]. Early recognition is crucial as delayed diagnosis can lead to rapid deterioration, particularly in patients who present with hemodynamic instability [12,13]. Maintaining a high index of suspicion is, therefore, crucial for timely interventions in such cases. 

CT scans are the cornerstone of diagnosing splenic rupture, as they not only confirm the diagnosis but also enable the evaluation of injury severity [13]. In cases where CT reveals high-density fluid within the abdomen, indicative of active bleeding, surgical intervention via laparotomy is usually necessary [12]. The urgency of such interventions underlines the importance of prompt and accurate imaging, particularly in cases where clinical signs may be subtle or atypical as they were in this case with right lower quadrant pain. 

Management of ASR follows similar principles established for traumatic splenic injuries, which are categorized using the American Association for the Surgery of Trauma (AAST) classification. Treatment decisions depend on factors such as the patient’s hemodynamic status, the extent of hemoperitoneum, and the volume of blood products required for stabilization [7]. While spleen preservation is prioritized in approximately 90% of traumatic splenic injuries, splenectomy is often unavoidable for certain hematological, malignant, or non-malignant causes of splenic rupture [10]. 

In instances requiring splenectomy, splenic autotransplantation has emerged as a potential strategy to mitigate the long-term immunological risks associated with asplenia, such as OPSI [14]. This technique involves reimplanting healthy splenic tissue into the abdominal cavity. The goal is to stimulate tissue regeneration, restore partial immunological function, and reduce the risk of severe infections in the absence of a functioning spleen [10]. 

Documented cases of successful autotransplantation include implantation sites such as the peritoneal cavity, retroperitoneum, abdominal wall muscles, liver, and omentum [15]. Of these, the omentum is particularly favorable due to its rich vascular supply, which supports tissue recovery and functionality [16]. However, autotransplantation is predominantly performed in controlled elective settings or in cases of traumatic splenic injury, where viable splenic tissue is more likely to be available and unaffected by underlying pathology [10]. 

In the case presented above, we explored the feasibility of splenic autotransplantation in non-traumatic splenic rupture. This approach relies on the identification of healthy splenic tissue during surgery. Studies have demonstrated that splenic tissue implanted into the omentum can regenerate, with both macroscopic and microscopic evidence of recovery [16]. Immunoglobulin recovery has been well documented in these cases, with scintigraphy studies further supporting the viability of the omentum as an implantation site due to its robust vascular network [16]. These findings highlight the potential for extending this approach to cases of ASR, provided that the underlying pathology does not compromise tissue viability. 

ASR is significantly less common than traumatic splenic rupture, yet it often necessitates more aggressive surgical management, with 84% of ASR cases requiring splenectomy compared to 50% of traumatic cases [1,9]. While infectious or inflammatory causes of ASR do not appear to increase mortality risk, neoplastic causes are associated with poorer prognoses ​[1]​. For patients with non-neoplastic ASR, autotransplantation may represent a viable treatment option offering a balance between managing acute surgical needs and preserving long-term immunological function. The risk of complications following autotransplantation is low, estimated at approximately 2-3%, which is comparable to other surgical procedures [16]. Expanding the application of this technique could improve outcomes in appropriately selected patients, particularly in cases where viable splenic tissue remains unaffected by underlying disease processes. 

## Conclusions

ASR is a rare, life-threatening condition that presents with varied symptoms, making diagnosis and management challenging. This case underscores the critical role of early CT imaging in ensuring accurate diagnosis and effective surgical planning, particularly in atypical presentations. While splenectomy is often necessary for unstable patients, the lifelong risk of OPSI highlights the importance of exploring alternatives like splenic autotransplantation. Notably, if healthy splenic tissue is identified in ASR cases, autotransplantation could be a viable option despite concerns about tissue viability. This approach shows potential in preserving partial immunological function, though further research is needed to evaluate its feasibility and refine management strategies.
